# Immediate postpartum intrauterine contraceptive device utilization and influencing factors in Addis Ababa public hospitals: a cross-sectional study

**DOI:** 10.1186/s40834-021-00148-7

**Published:** 2021-02-02

**Authors:** Yohannes Fikadu Geda, Seid Mohammed Nejaga, Mesfin Abebe Belete, Semarya Berhe Lemlem, Addishiwet Fantahun Adamu

**Affiliations:** 1 Department of Midwifery, Wolkite University, Wolkite, Ethiopia; 2 College of Health Science, Black Lion Specialized Hospital, Addis Ababa University, Addis Ababa, Ethiopia; 3 School of Nursing and Midwifery, Addis Ababa University, Addis Ababa, Ethiopia

**Keywords:** Immediate postpartum, Intrauterine contraceptive device, Contraception, Addis Ababa

## Abstract

**Background:**

Postpartum intrauterine device (PPIUCD) utilization remains very low in Ethiopia beside high levels of unmet need for postpartum family planning even if nongovernmental organizations efforts to promote its use. This study investigates immediate PPIUCD utilization and influencing factors.

**Methods:**

Institution based cross-sectional study was conducted on public hospitals of Addis Ababa city. All public hospitals which have PPIUCD service were included and systematic random sampling technique was used to select 286 participants. Data were entered using Epi Data and exported to SPSS for analysis. Bivariate and multivariate logistic regression analysis was used to determine the effect of independent variables on immediate PPIUCD utilization. Variables which have *P*-value< 0.2 on bivariate analysis were candidate for multivariate analysis. Variables which have *P*-value ≤0.05 on multivariate analysis was considered as statistically significant.

**Results:**

Utilization of immediate PPIUCD among participants who gave birth in Addis Ababa public hospitals was 26.6% (95%CI: 21.3, 31.8). Eighty one percent respondents occupation was housewife were (AOR = 0.19, 95%CI: 0.06, 0.67) less likely to utilize PPIUCD compared to those who have personal job. In the other hand respondents who have discuss about PPFP with their partner were 1.21times (AOR = 1.21, 95%CI: 1.14, 25.67) more likely to utilize PPIUCD compared to those who never discuss. Contrarily 81% of respondents who need partner approval were (AOR = 0.19, 95%CI: 0.05, 0.79) less likely to utilize PPIUCD compared to those who doesn’t need approval. Respondents who have been counseled about PPIUCD were 1.13 times (AOR = 1.13, 95%CI: 1.10, 2.21) more likely to utilize PPIUCD compared to those who were not counseled. Similarly respondents who have good knowledge about PPIUCD were 7.50 times (AOR = 7.50, 95%CI: 4.06, 9.31) more likely to utilize PPIUCD compared to those who have poor knowledge.

**Conclusion:**

This study verifies that immediate PPIUCD utilization is high compared to other studies. Having a housewife occupation and necessity of partner approval to utilize PPIUCD have negative influences, whereas spousal discussion about PPIUCD, counseled during pregnancy and having good knowledge have positive influences on PPIUCD utilization. Therefor empowering women by the government and other organizations working on maternal health will advance immediate PPIUCD utilization.

## Background

Postpartum family planning (PPFP) is defined as the use of family planning in the first 12 month following birth [1, 2]. Fertility after birth can return as soon as 45 days after giving birth for women who are not breastfeeding [3, 4] and it can also occur before menses is resumed on those who don’t feed breast exclusively [5].

World Health Organization (WHO) recommends spacing pregnancies by at least 24 months [6]. However, unmet need for family planning is high in the postpartum period, ranging from 32 to 62% in low and middle- income countries [3, 7, 8]. Because of unmet need, unintended pregnancy is common in postpartum period [9, 10]. Beside this closely spaced pregnancies are expected to have adverse maternal, perinatal and infant outcomes [11, 12].

Postpartum intrauterine contraceptive device (PPIUCD) is one of the contraceptive methods which is safe and highly effective, reliable and long acting contraceptive [13, 14]. The effectiveness of intrauterine device (IUCD) as a contraceptive method is approximately 99.2 to 99.8% within the first year of use, which is better than other shorter-term reversible contraceptive methods [15, 16].

PPIUCD is an acceptable contraception with fewer complications [17, 18]. PPIUCD inserted just after 10 min of placental delivery is a safe, effective, and efficient method of meeting women’s need for long-acting but reversible method of contraception [19, 20]. It can be placed after abortion, vaginal delivery and during caesarian section [21].

In Ethiopia PPIUCD intervention on selected public health facility is started on 2014 even if it is not widely utilized [6]. Intrauterine device is the least utilized modern contraceptive method in Ethiopia [22]. Reasons for low utilization range from limited provision of IUCDs to a lack of staff trained in providing family planning counseling and services to pre-existing biases against IUCDs among both providers and the general public [19, 23–26].

Unmet need of family planning among married women in Ethiopia is 22% [22]. In addition to this 17% pregnancies were mistimed, and 8% of pregnancies were unwanted [27]. Increasing access to effective postpartum intrauterine contraceptive methods can reduce the risk of unintended pregnancy and short inter-birth intervals [28].

Postpartum unintended pregnancy is an important public health challenge in Ethiopia. Moreover there are very few studies conducted on PPIUCD in low resource setting and it is an emerging services. Understanding the level of PPIUCD utilization will provide information that can be used by policy makers and other stakeholders to improve service delivery of PPIUCD. There for this study aimed to assess immediate PPIUCD utilization and influencing factors in Addis Ababa public hospitals.

## Methods

### Study setting and design

This study was conducted in public hospitals of Addis Ababa. Addis Ababa is the capital city of Ethiopia. The city has 10 sub-cities and located at the geographical center of the country. According to national census annual population growth rate of Addis Ababa was 2.1% between 1994 and 2007 and current total projected population size of 4,005,597, out of these female population accounted 52%. Women of reproductive age group among the total population are 947,855 [29].

Addis Ababa city has 13 public hospitals distributed throughout 10 sub cities. The public hospitals in the city are Black Lion Specialized Hospital (BLSH), St Paul Hospital Millennium Medical College (SPHMMC), Amanuel Hospital (AmH), Alert Hospital (AlH), St Peter Hospital (SPH), Police Hospital (PH), Armed Force Hospital (AFH), Zewditu Memorial Hospital (ZMH), Menilik II Memorial Hospital (MMH), Ras-Desta Memorial Hospital (RDMH), Yekatite-12 Hospital (YH), Tirunesh Beijing Hospital (TBH) and Gandhi Memorial Hospital (GMH). On the selected hospitals postnatal ward institution based crossectional study was conducted from August 25–September 30, 2019.

### Study subject

All postpartum women who gave birth in Addis Ababa public hospitals were the source population. Postpartum women who have had fulfilled WHO eligibility criteria for insertion of immediate postpartum IUCD were during the study period were included. Those who have high Fever during labor and delivery, having active sexual transmitted diseases (STD) or other lower genital tract infection or high risk for STD, ruptured membrane for more than 24 h prior to delivery, known uterine abnormalities, unresolved post-partum hemorrhage or postpartum uterine atony requiring use of additional oxytocic drugs were excluded from the study in accordance with WHO exclusion criteria [30].

### Sample size determination, sampling technique and procedures

The sample size was determined by using single population proportion formula. By considering 5% margin of error, 95% confidence interval (CI), 21.6% proportion of immediate PPIUCD practice from a study done in Sidama Zone, southern Ethiopia [31], and none response rate of 10% making final sample size of 286.

**Fig. 1:**
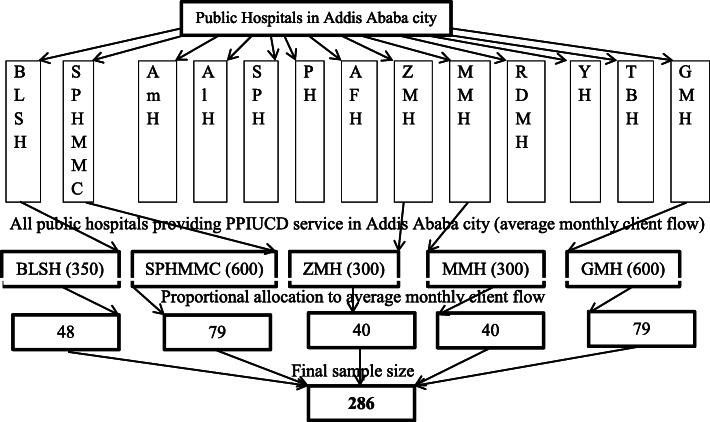
sampling procedures of study participants in Addis Ababa public hospitals, Addis Ababa, August 25 to September 30, 2019

From the total public hospitals in Addis Ababa city all hospitals providing PPIUCD service were included in this study. Hospitals providing PPIUCD service are BLSH, SPHMMC, ZMH, MMH, and GMH. To select study participants, the total sample size was allocated proportionally to each included hospital based on the average number of monthly delivery service. Postpartum women’s in each included hospitals were selected using systematic random sampling technique. To select individual participant, order of delivery record book was used and interval of selection was calculated based estimated average monthly client flow to each hospital (Fig. 1).

### Variables

Immediate Postpartum intrauterine contraceptive utilization was dependent variable of this study. Socio-demographic characteristic (age, marital status, occupation, educational status, religion); knowledge, attitude, obstetric characteristics (birth interval, decision to use family planning, number of birth and children, planned/unplanned Pregnancy); health service related (numbers of Antenatal care visits, Family Planning Counseling during Antenatal care and delivery setting, previous Family Planning experience, mode of delivery) were independent variables.

### Operational definitions

#### Immediate PPIUCD

An IUCD that can be inserted post placental, intra cesarean and spontaneous vaginal delivery within 48 h of delivery.

#### Utilization of PPIUCD

Postpartum women who have used postpartum intrauterine contraceptive device.

#### Good knowledge

A score of greater than or equal to mean (correct answers for 5 or more out of 10) of knowledge assessment questions.

#### Poor knowledge

A score of less than mean of the knowledge assessment questions.

#### Favorable attitude

A score of greater than or equal to mean (correct answers for 2.5 or more out of 5) of attitude assessment questions.

#### Unfavorable attitude

A score of less than mean of the knowledge assessment questions.

### Data collection tools and procedures

A pretested structured interviewer administered questionnaire consisting of items with pre-coded response categories was used. The questionnaire was adopted from EDHS 2016 [27] and reviewing literatures.

The tool has four sections: The first section consists of socio demographic characteristics, second section was about obstetrics related Practice, third section assesses knowledge, fourth section was about attitude and last section was about PPIUCD utilization and related characteristics of study participants. The questionnaire was designed in English and translated in to local Amharic language and then translated back to English by translators for consistency.

Data were collected by face to face interview. Five BSc midwifes for data collection and one MSc midwife for supervision were recruited. One day data collection training was given to data collectors and supervisors on the objectives, benefits of the study, individual‘s right, informed consent and techniques of the interview.

Before starting the actual data collection to assure the data quality, high emphasis was given to designing data collection instrument. First the questionnaire was pre-tested on 10% of sample size, 26 postpartum women in Teklehaymanot general hospital. After pre-test further adjustments on the tool was made to improve clarity, understandability, and simplicity of the messages.

All of the questionnaires were checked for completeness and accuracy before, during and after the period of data collection. Throughout the course of the data collection, interviewers were supervised. Regular meetings were held between the data collectors and the principal investigator to discuss on challenges and solutions of procedures. The collected data was again reviewed and checked for completeness before data entry. Data entry format template was prepared and programmed by principal investigator.

### Data analysis

Data were entered using Epi Data version 4.2, and exported to statistical package of social sciences (SPSS) version 24.0 for analysis. Descriptive statistics were computed to describe study variables in relation to the population. Bivariate and multivariate logistic regression was used to determine the effect of independent variables on immediate PPIUCD utilization. Variables which have *P*-value< 0.2 on bivariate analysis were selected as a candidate for multivariate analysis. Hosmer-Lemeshow goodness-of-fit test was used to check fitness of the model, and it was best fitted with *P* = 0.84. Variables which have *P*-value ≤0.05 on multivariate analysis was considered as statistically significant factors influencing immediate PPIUCD utilization. Finally, results were compiled and presented using texts and tables.

## Results

A total of 286 included respondents were participated in this study, which makes response rate of 100%. About half of the respondents 141(49.3%) belongs to age category of 25–29 year with mean age of 28.0 and standard deviation of 4.69. Majority of the study participants 271(88.1%) were married (Table 1).

**Table 1:** Socio-demographic characteristics of post-partum women in Addis Ababa public hospitals, Addis Ababa, August 25 to September 30, 2019-(*N* = 286)

Variable	Frequency	Percent (%)
**Age (years)**		
≤ 19	4	1.4
20–24	58	20.3
25–29	141	49.3
30–34	47	16.4
Above 35	36	12.6
**Marital Status**		
Married	252	88.1
Divorced	20	7.0
Unmarried	6	2.1
Widowed	8	2.8
**Level of education**		
No formal education	21	7.3
Primary (1–8)	90	31.5
Secondary (9–12)	104	36.4
College and above	71	24.8
**Religion**		
Orthodox	124	43.4
Muslim	108	37.8
Protestant	46	16.1
Catholic	8	2.8
**Occupation**		
Housewife	106	37.1
Government employee	28	9.8
Private employee	51	17.8
Daily laborer	20	7.0
Self employed	56	19.6
Merchant	25	8.7

### Obstetrics related practice of study participants

The mean age at first marriage was 20 year with standard deviation of 3.92. More than two third of study participants 210(73.4%) had at least one birth. About 81(37.2%) of the participants had a birth spacing of above 36 months (Table 2).

**Table 2:** Obstetric Characteristics of the participants who gave birth in Addis Ababa public hospitals, Addis Ababa, August 25 to September 30, 2019-(*N* = 286)

Variable	Frequency	Percent (%)
**Age at first marriage**		
≤ 18	76	26.6
19–20	86	30.1
21+	124	43.4
**Number of Birth**		
1–2	210	73.4
3–4 children	68	23.8
5 and above	8	2.8
**Age of last child (months)** (*n* = 218)		
< 24	62	28.4
24–36	75	34.4
> 36	81	37.2
**Plan to have another child**		
Yes	173	60.5
No	72	25.2
Undecided	41	14.3
**Time to have another child in the future (months)**(*n* = 173)		
Less than 24	31	17.9
24–36	52	30.1
Above 36	90	52.0
**Number of alive children**		
None	4	1.4
1–4	210	73.4
5 and above	72	25.2
**Number of children want to have in your life**		
≤ 3	106	37.1
≥ 4	180	62.9
**present birth planned**		
Yes	212	74.1
No	74	25.9
**Decision on the use of modern FP**		
Mainly respondents	64	22.4
Mainly Husband	44	15.4
Jointly decision	178	62.2
**Discussed on family planning methods with partner during pregnancy**		
Yes	133	46.5
No	153	53.5
**Partner approves of family planning use**		
Yes	113	39.5
No	173	60.5

### Knowledge of participants about postpartum intrauterine contraceptive device

This study showed that more than two third of the study participants 179(68.9%) of them responded that they have heard IUCD can be inserted immediately after delivery. From the study participants 92.3% of them answered PPIUD can be removed at any time you wish, followed by 75.5% who says PPIUCD is inserted free of charge in Ethiopia and 73.4% of them responded PPIUCD does not cause cancer (Table 3).

**Table 3:** knowledge of postpartum women about PPIUCD in Addis Ababa public hospitals, Addis Ababa, August 25 to September 30, 2019-(*N* = 286)

Questions	Responses				Correct responses	Percent correct
	TRUE		FALSE			
	N	%	N	%		
PPIUD can prevent pregnancies for more than 10 years.	176	61.5	110	38.5	TRUE	61.5
PPIUD is not appropriate for females at high risk of getting STIs.	147	51.4	139	48.6	FALSE	48.6
PPIUD has no interference with sexual intercourse or desire.	148	51.7	138	48.3	TRUE	51.7
PPIUD is immediately reversible (become pregnant quickly when removed).	182	63.6	104	36.4	TRUE	63.6
PPIUD does not cause cancer.	210	73.4	76	26.6	TRUE	73.4
PPIUD can be used by breast feeding mothers.	202	70.6	84	29.4	TRUE	70.6
PPIUD may cause changes in bleeding pattern.	196	68.5	90	31.5	TRUE	68.5
PPIUD can be used by HIV positive patients doing well on treatment.	117	40.9	169	59.1	TRUE	40.9
PPIUD is inserted free of charge in Ethiopia.	216	75.5	70	24.5	TRUE	75.5
PPIUD can be removed at any time you wish.	264	92.3	22	7.7	TRUE	92.3

### Participants attitude about postpartum intrauterine contraceptive device

The respondents were asked to reflect their opinion on a serious of questions concerning to attitude towards post-partum IUCD. The Likert scale with scores ranging from 1 = disagree to 3 = agree was used to measure their attitude (Table 4).

**Table 4:** Attitude of postpartum women about PPIUCD utilization in Addis Ababa public hospitals, Addis Ababa, August 25 to September 30, 2019-(*N* = 286)

Questions	Frequency	Percent (%)
**Insertion and removal of PPIUD is highly painful**		
Agree	234	81.8
Not sure	38	13.3
Disagree	14	4.9
**PPIUCD doesn’t move through the body after insertion**		
Agree	202	70.6
Not sure	60	21.0
Disagree	24	8.4
**PPIUCD does not interfere with sexual intercourse**		
Agree	236	82.5
Not sure	34	11.9
Disagree	16	5.6
**PPIUD is very effective at pregnancy prevention**		
Agree	184	64.3
Not sure	84	29.4
Disagree	18	6.3
**PPIUCD can harm a woman’s womb**		
Agree	184	64.4
Not sure	80	28.0
Disagree	22	7.7

### PPIUCD utilization and related characteristics of study participants

The study participants ever used family planning methods was 220(76.9%). Current use of PPIUCD among study participants were 76(26.6%). All of the study participants were attended ANC visits during current pregnancy and among the women 87(30.4%) had received family planning counseling (Table 5).

**Table 5:** PPIUCD utilization and related characteristics of participants who gave birth in Addis Ababa public hospitals, Addis Ababa, August 25 to September 30, 2019-(*N* = 286)

Variable	Frequency	Percent (%)
**Ever used family planning methods previously**		
Yes	220	76.9
No	66	23.1
**Family planning use before recent pregnancy (*n*** **= 220)**		
Yes	180	81.8
No	40	18.2
**Method used (*n*** **= 220)**		
Natural Family Planning	7	3.2
IUCD	42	19.1
Implanon	62	28.2
Injectable	76	34.5
Pills	33	15.0
**Currently using PPIUCD**		
Yes	76	26.6
No	210	73.4
**Reason not to use PPIUCD (*n*** **= 210)**		
wanted to have another child	31	14.8
Not think I could be pregnant	30	14.3
Religion Prohibition	14	6.7
Husband disapproves	13	6.2
Afraid of side effects	78	37.1
Afraid of becoming infertile	24	11.4
Use later when menstruation begins	20	9.5
**Mode of delivery**		
SVD	197	68.9
Vacuum/ Forceps delivery	33	11.5
C/S	56	19.6
**Have antenatal care follow up**		
Yes	286	100
**Number of antenatal care visits**		
1 visit	2	0.7
2–3 visits	42	14.7
4 visits and above	242	84.6
**PPIUCD counseling in the health facilities**		
Yes	87	30.4
No	199	69.6
**Time of PPIUCD counseling (*n*** **= 87)**		
During ANC follow up	69	79.3
During labor	4	4.6
After delivery	14	16.1

### Influencing factors of PPIUCD utilization

On bivariate analysis age, level of education, occupation, time to have more children, discussion about family planning with partner, needs partner approval to use PPFP, counseled about PPIUCD and knowledge were candidates for multivariate analysis. From this variables Occupation, discuss about FP with partner, needs partner approval to use FP, counseled about PPIUCD and knowledge were remain statistically significant predictors to utilize PPIUCD (Table 6).

**Table 6:** PPIUCD utilization predictors among participants who gave birth in Addis Ababa public hospitals, Addis Ababa, August 25 to September 30, 2019-(*N* = 286)

Variables/Category	PPIUCD		COR(95% CI)	AOR(95% CI)
	Utilized N (%)	Not utilized N (%)		
Age				
15–24	8 (10.5)	54 (25.7)		
25–34	54 (71.1)	134 (63.8)	0.37 (0.16, 0.82)	0.29 (0.08, 1.14)
> 35	14 (18.4)	22 (10.5)	0.23 (0.08, 0.63)	0.51 (0.09, 2.76)
Level of education				
No formal education	2 (2.6)	19 (9.0)	5.49 (1.18, 25.47)	1.87 (0.02, 34.92)
Primary (1–8)	7 (9.2)	83 (39.5)	6.85 (2.76, 17.02)	1.61 (0.09, 4.15)
Secondary (9–12)	41 (53.9)	63 (30.0)	0.89 (0.48, 1.66)	0.62 (0.57, 4.62)
College and above	26 (34.2)	45 (21.4)	1	1
Occupation				
Housewife	22 (28.9)	84 (40.0)	0.76 (0.69, 2.53)	0.19 (0.06, 0.67)*
Employed^a^	28 (36.8)	51 (24.3)	0.63 (0.33, 1.19)	0.52 (0.15, 1.82)
Personal job^b^	26 (34.2)	75 (35.7)	1	1
Time to have more child				
< 24 months	13 (29.5)	18 (14)	0.39 (0.17, 0.94)	3.56 (0.94, 13.81)
24–36 months	11 (25)	41 (31.8)	1.06 (0.46, 2.44)	0.98 (0.33, 2.96)
> 36 months	20 (45.5)	70 (54.3)	1	1
Discuss about PPFP with partner				
Yes	37 (48.7)	116 (55.2)	1.30 (0.76, 2.20)	1.21 (1.14, 25.67)*
No	39 (51.3)	94 (44.8)	1	1
Needs partner approval to use PPFP				
Yes	51 (67.1)	122 (58.1)	0.68 (0.39, 1.18)	0.19 (0.05, 0.79)*
No	25 (32.9)	88 (41.9)	1	1
Counseled about PPIUCD during pregnancy				
Yes	25 (48.7)	88 (23.8)	1.16 (1.12, 2.01)	1.13 (1.10, 2.21)**
No	39 (51.3)	160 (76.2)	1	1
Knowledge about PPIUCD				
Poor	66 (86.8)	72 (34.3)	1	1
Good	10 (13.2)	138 (65.7)	12.65 (6.14, 26.08)	7.50 (4.06, 9.31)***

* = *P* < 0.05, ** = *P* < 0.01, *** = *P* < 0.001, ^a^ = gov’t & private employee, ^b^ = merchant, self-employee and daily laborer

From the respondents 81% of those whose occupation is housewife were (AOR = 0.19, 95%CI: 0.06, 0.67) less likely to utilize PPIUCD compared to those who have personal job. In the other hand respondents who have discuss about PPFP with their partner were 1.21times (AOR = 1.21, 95%CI: 1.14, 25.67) more likely to utilize PPIUCD compared to those who never discuss. Contrarily 81% of respondents who need partner approval to use PPFP were (AOR = 0.19, 95%CI: 0.05, 0.79) less likely to utilize PPIUCD compared to those who doesn’t need approval. Respondents who have been counseled about PPIUCD were 1.13 times (AOR = 1.13, 95%CI: 1.10, 2.21) more likely to utilize PPIUCD compared to those who were not counseled. Similarly respondents who have good knowledge about PPIUCD were 7.50 times (AOR = 7.50, 95%CI: 4.06, 9.31) more likely to utilize PPIUCD compared to those who have poor knowledge (Table 6).

## Discussion

In this study immediate PPIUCD utilization and influencing factors among participants who gave birth in Addis Ababa public hospitals was assessed. In accordance with immediate PPIUCD utilization knowledge and attitude of participants was studied as an independent predictors. In addition, effect of socio-demographic, obstetric and related factors over PPIUCD utilization was studied.

Immediate PPIUCD utilization among participants who gave birth in Addis Ababa public hospitals was 26.6% (95%CI: 21.3, 31.8). Similar figure of PPIUCD utilization was reported in Sidama region, Ethiopia [31] and another study in Rwanda [32]. This is higher than the national report of Ethiopian mini DHS 2019 which is 2% [22]. Similarly this result is higher than a study done in Sri Lanka 3.4% [11].

This difference might be due to proportion of women receiving postnatal care in Addis Ababa is higher which is 74% unlike other part of the country like 10% in Somali [22]. This might also be due to peoples living in Addis Ababa (capital city of the country) having better route of information. Additionally peoples living in Addis Ababa have different religions whereas Somali region is dominated by Muslim religion followers and the religion prohibits family planning utilization.

From the respondents 81% of those whose occupation is housewives were less likely to utilize PPIUCD compared to those who have personal job. Similarly a study conducted in Adaba town [33], Bahirdar town [34] and Janamora district [35] house wives were less likely to utilize long acting family planning compared to daily laborers.

This might be due to women’s working outside home may have economic difficulties to have additional family member. Additional reasons might be house builders (house wives) have other source of income unlike daily laborers and others.

Respondents who discuss about PPFP with their partner were more likely to utilize PPIUCD compared to those who never discuss. Consistently a study conducted in Bahirdar town explains that women’s who have spousal discussion were utilizing long acting family planning [34].

Respondents who need partner approval to use PPFP less likely to utilize PPIUCD compared to those who don’t need approval. Similarly a study conducted in Gamogofa zone public health facilities states that odds of mothers who have partner support for IUCD insertion were more likely to utilize PPIUCD than those do not have partner support [10].

This might be due to women’s usually depends on their husband’s decision in Ethiopia, even though government and different nongovernmental organizations were working on empowering women. In addition to this it might also be due to take care of their marriage and families from unresolvable quarrels and to prevent divorce. Another description might be decisions made jointly with agreement of both couples will have better outcome since issue of family planning is not only the concern of one partner.

In this study participants who have been counseled about PPIUCD were more likely to utilize PPIUCD compared to those who were not counseled. Similarly a study conducted in Sidama region [31] and Bahirdar town [36] shows counseled clients were dedicated to utilize PPIUCD.

Another clustered randomized data in Tanzania explains that giving women informational materials on PPIUCD and counseling after admission for delivery are likely to increase the proportion of women choosing PPIUCD [19]. In the same manner a study in India assures that counseling in the antenatal period was a key point in increasing acceptance of PPIUCD [37].

This might be because women who are received family planning counseling during ANC and PNC might be highly motivated to use PPIUCD. This might also be due to counseling can solve traditional attitudes and myths thinking that PPIUCD is bad. In the other hand counseling can made clients to improve their knowledge about the methods they are going to use. Result of this study verifies that clients who have good knowledge were utilizing PPIUCD.

Respondents who have good knowledge about PPIUCD were more likely to utilize PPIUCD compared to those who have poor knowledge. In the same manner a study conducted in Janamora district [35] and Bahirdar town [34] women who had good knowledge were utilize PPIUCD compared to those who had poor knowledge.

Consistently a study conducted in India states that clients who have good knowledge have better experience of PPIUCD [38]. In the other hand this study describes that 63.6% of participants scored mean and above the mean of attitude assessment questions, and considered as having a favorable attitude towards PPIUCD; attitude was not statistically a significant factor to utilize PPIUCD.

### Strength and limitation of the study

This study well presents residence of Addis Ababa city, their stance of utilizing immediate PPIUCD and identify the bottle neck factors for the service. Contrary to this it was conducted in Addis Ababa city (capital city of the country) public hospitals; hence the findings might not be adequately reflected the entire population and other cities of Ethiopia.

## Conclusion

This study concludes that PPIUCD utilization among Addis Ababa city public hospital was high compared to other studies. Participants whose occupation was housewife and who needs partner approval to utilize PPIUCD were less likely to utilize PPIUCD compared to their counter parts. Whereas participants who have discussed about PPIUCD with their spouse, have counseled about PPIUCD during their pregnancy and participants who have good knowledge were more likely to utilize PPIUCD compared to their counter parts. Based on this result the following recommendations were given.

- Housewife’s need to be supported by Addis Ababa health office and health extension workers to work on PPIUCD utilization by delivering appropriate information.
- Discussion about family planning in the community at large in Ethiopia is not common. There for discussion with partner about family planning need to be a culture and should be encouraged by health professionals working on maternal health services.
- Good counseling can address client myths and misconceptions about the PPIUCD there for health professionals delivering this service should give emphasis for it. Each facilities working on this service should integrating postnatal care with post-partum family planning counseling.

## Data Availability

The datasets supporting result of this study is available from the corresponding author on reasonable request, but it is not available in public in favor of participant’s confidentiality.

## Abbreviations

AFH: Armed Force Hospital
AlH: Alert Hospital
AmH: Amanuel Hospital
ANC: Antenatal Care
AOR: Adjusted Odds Ratio
BLSH: Black Lion Specialized Hospital
CI: Confidence Interval
FP: Family Planning
GMH: Gandhi Memorial Hospital
MMH: Menilik II Memorial Hospital
PH: Police Hospital
PNC: Postnatal Care
PPFP: Post Partal Family Planning
PPFP: Postpartum Family Planning
PPIUCD: Post Partal Intrauterine Contraceptive Device
RDMH: Ras-Desta Memorial Hospital
SPH: St Peter Hospital
SPHMMC: St Paul Hospital Millennium Medical College
SPSS: Statistical Package of Social Sciences
STD: Sexual Transmitted Diseases
TBH: Tirunesh Beijing Hospital
WHO: World Health Organization
YH: Yekatite-12 Hospital
ZMH: Zewditu Memorial Hospital
